# Proposed Algorithm for Integrated Management of TB-SARS-CoV-2 Co-Infection in a TB-Endemic Country

**DOI:** 10.3390/tropicalmed7110367

**Published:** 2022-11-10

**Authors:** Ni Made Mertaniasih, Soedarsono Soedarsono, Tiffany Tiara Pakasi, Zakiyathun Nuha, Manabu Ato

**Affiliations:** 1 Department of Medical Microbiology, Faculty of Medicine, Universitas Airlangga, Surabaya 60131, Indonesia; 2 Sub-Pulmonology Department of Internal Medicine, Faculty of Medicine, Hang Tuah University, Surabaya 57600, Indonesia; 3 Tuberculosis Laboratory, Institute of Tropical Disease, Universitas Airlangga, Surabaya 60115, Indonesia; 4 National TB Program, Directorate of Communicable Disease Prevention and Control, Ministry of Health Indonesia, Jakarta 10710, Indonesia; 5 Department of Mycobacteriology, Leprosy Research Center, National Institute of Infectious Diseases, Tokyo 162-0052, Japan

**Keywords:** tuberculosis, COVID-19, integrated management, algorithm, respiratory infection

## Abstract

Tuberculosis (TB) and COVID-19 have become significant health problems globally, especially in countries with high prevalence. Therefore, this research aims to examine all possibilities and predict the impact of TB-SARS-CoV-2 co-infection to anticipate the cascade effect of both diseases in all sectors. The conceptual strategy of the algorithm in TB-COVID-19 is needed to create an integrated management system. It includes the stages of early detection with accurate and effective methods, as well as the synchronization of TB-COVID-19 health services, starting from primary health facilities to secondary and tertiary referral centers. The algorithm in TB-COVID-19 is crucial to prepare future strategies for PTB co-infection viral respiratory infections other than SARS-CoV-2, ILI, ARI, and SARI. Since the implementation involves all health services, there is a need to integrate the governance of TB-COVID-19 and other comorbidities in good health services based on research and multicentre design.

## 1. Introduction

Tuberculosis (TB) remains a leading menace in infectious disease, and COVID-19 has become a global pandemic since it was announced by World Health Organization (WHO) on 11 March 2020. This is a double burden for TB-endemic countries such as India, China, and Indonesia [1,2,3]. Furthermore, there has been an increase in TB-SARS-CoV-2 co-infection cases, which are reported as rare [4,5,6,7]. Due to the potentially worse outcome of co-infection, a comprehensive and intensive investigation is required from all sectors.

The understanding of all possibilities and predicting the effect of TB-COVID-19 is critical to anticipate the cascade effect of both diseases in all sectors and other communicable diseases such as Influenza-Like Illness (ILI), Severe Acute Respiratory Infections (SARI), and Acute Respiratory Infection (ARI). It is necessary to reinforce monitoring and evaluation of the implementation of TB control programs integrated with other communicable diseases. Awareness of the importance of overcoming problems encourages various strategic efforts to establish integrated management systems in the health systems in the future, which elevates the role of various aspects, including multicentre collaborations. Moreover, consistent research is also needed to design an efficient management system strategy for serious communicable diseases that can be implemented globally.

TB is a complicated infectious disease; therefore, several cases, such as antimicrobial resistance and Latent TB Infection (LTBI), need immediate solutions [8,9]. However, many health policies have weakened implementation due to the pandemic. The similarity of features in respiratory tract infections also complicates identification, delaying the process of handling and treatment [10,11]. This condition is exacerbated by the high population and TB cases in low-middle-income countries such as India, Indonesia, and Pakistan [2,12].

Since COVID-19 was declared a pandemic, communities and governments were mobilized to contain the further spread and reduce the effect on populations, health structures, and economies. This is because COVID-19 affects the vulnerable population and those affected by communicable diseases. For example, the stimulating effects of the pandemic will result in a global spread of a TB epidemic and the risk of its biological effects, such as the interaction of two diseases [13,14,15]. Therefore, this research was carried out to create the best strategy to implement integrated management systems and anticipate the complex problem in emerging or re-emerging communicable diseases for the global goal of suitable environments, good health, and welfare.

## 2. Occurrence of TB-SARS-CoV-2 Co-Infection

Data from meta-analysis and a literature study with extensive publication searches from various countries found 86 cases from 36 studies of TB-SARS-CoV-2 co-infection [6]. TB and COVID-19 have symptoms that tend to be the same because they attack the lungs [13,16,17]. Analytical data of lung imaging features showed the 10 most common similarity features respectively, cavities (32.58%), infiltrates (31.46%), ground-glass opacity (19.1%), nodules (16.85%), pleural effusion (11.24%), fibrosis (12.36%), patchy shadows (8.99%), consolidation (8.99%), military lesions (5.62%), and reticules (5.62%), and the most significantly different in laboratory examination is leucocyte count. The survivor showed a lower leucocyte count than the non-survivors (8.015 [4.8–8.97] vs. 12.9 [10.5–16.73] × 109/L, *p* = 0.007) [6]. A cohort study stated that patients with TB-COVID-19 have both higher mortality rates and severe symptoms than those with only COVID-19 [6,18]; data from a pooled study comparing the TB-COVID-19 group and non-TB group regarding odds ratios (ORs) showed the percentages of deaths was 3.46% (822/23,732) in the overall group, 5.69% (123/2161) in the TB-COVID-19 group, and 3.24% (699/21,571) in the non-TB group; data from WHO also showed the increased number of TB death between 2019–2020. An important factor that affected the mortality rate in TB cases is the lower TB detection rate which decreased by 18% during the pandemic [2,6]. Although the number of TB-COVID-19 cases is low, the severity and high mortality rate remain a threat [19,20,21]. It was also discovered that TB management is affected by the pandemic, such as decreasing the number of TB cases findings in several countries [2,22,23,24]. Meta-analyses show that TB-COVID-19 patients aged >65 years have a higher risk of death and comorbidity rate [6,25]. Some evidence indicates that gender also affects the risk of these diseases, where males have a more vulnerable risk than females [6,18] due to high cases of TB [26,27]. An important factor that affected the mortality rate in TB cases is the lower TB detection rate which decreased by 18% during the pandemic [2,6]. Although the number of TB-COVID-19 cases is low, the severity and high mortality rate remain a threat [19,20]. It was also discovered that TB management is affected by the pandemic, such as decreasing the number of TB cases findings in several countries [2,22,23,24]. Meta-analyses show that TB-COVID-19 patients aged >65 years have a higher risk of death and comorbidity rate [6,25]. Some evidence indicates that gender also affects the risk of these diseases, where males have a more vulnerable risk than females [6,18] due to high cases of TB [26,27].

## 3. Tuberculosis and COVID-19 Integrated Management

The COVID-19 pandemic will impact TB management when the focus of attention is shifted to response and dealing with the prolonged spread of infection [28,29]. Therefore, integrated TB-COVID-19 management is needed to minimize the effect of the pandemic on the TB program. Due to the similarities of both diseases [30], integrated TB-COVID-19 management needs to be constructed quickly [17,31]. This can be developed with monitoring, surveillance systems, infrastructure, robust programs that have years of advanced TB end programs in many countries, and advanced diagnostic tools [13,32].

A standard algorithm that includes early detection stages with accurate and effective methods is required to determine the management of patients with suspected TB-COVID-19 and prevent disease progression from becoming severe. It also involves the synchronization of TB-COVID-19 health services, starting from primary health facilities, secondary as well as tertiary referral centers, and preparing future strategies for co-infection of pulmonary tuberculosis (PTB) respiratory virus infections other than SARS-CoV-2, ILI, ARI, and SARI [33,34,35,36,37]. Therefore, a standard algorithm is an integrated TB-COVID-19 case management activity based on the mechanism of lower respiratory tract infection, starting with the transmission of agents via airborne and considering similar factors that attack the primary lung tissue and interfere with the host immunity [13,38]. The implementation of the TB-COVID-19 management program needs consideration in many sectors. These include COVID-19 screening, testing, and adherence support of all HIV, TB, MDR-TB, other patients with chronic disease, and severe acute respiratory tract infection ILI, ARI, and SARI [39,40,41,42].

The U.S. Agency for International Development (USAID) statement regarding the guidelines for implementing the Tuberculosis Contact Investigation Program (PI-TBCI) stated that it is important to implement TBCI integrated with COVID-19 contact tracing at the household, health facility, and broader community levels. The integration approach in detecting TB-COVID-19 cases can increase efficiency in using human resources, strengthen the community, and sustain the implemented disease control and prevention strategy. The strategy for health system services includes the community-level early warning system, the primary healthcare early detection point, and a nationwide diagnostic network. To effectively locate active cases, a systematic contact investigation in households and among social contacts of active disease processes is required as the crucial guideline of TBCI and COVID-19 screening in households. This is related to the triage unit of health facilities and an integrated referral system. Based on the prepared guidelines, especially the community approach, the mapping of high-burden sites and diagnostic capacity mapping is recommended to sustain community-based treatment monitoring and digital adherence interventions [43]. This is because active case finding with a community approach is the potential strategy for sustained TB programs in the COVID-19 pandemic. Therefore, health facilities need consistency to anticipate the importance of this approach in terms of patients seeking care [44,45].

Meaningful actions must be implemented to cope with the complex disease problem that can spread quickly. Furthermore, controlling fear and stigma in the community regarding infectious diseases is no less critical. It starts from the One Health approach launched by WHO, which improves the management system and stakeholders in the health sector, from primary healthcare to referral hospitals [12,46,47]. LTBI problems were also reported to positively impact the severity of COVID-19 infection. Previous reports also showed that the role of LTBI can reduce COVID-19 mortality. However, LTBI policy guidelines must be taken seriously due to the high activation probability [48,49,50].

## 4. Algorithm TB-COVID-19 Design

A comprehensive framework for managing diseases, especially pulmonary infection, is essential to prevent contagious spreading in the community or population. An algorithm is an indispensable tool for standardizing the efficient management of patients with pulmonary disease, especially TB-COVID-19 co-infection.

Therefore, the TB-COVID-19 algorithm is based on TB incidence, history, contacts, and clinical manifestations. These include signs and symptoms, abnormal laboratory results and CXR abnormalities, active infiltrate and chronic infection characteristics, other comorbidities, and vulnerable people. Decision-making methods are also required to identify the causative *Mycobacterium tuberculosis* (MTB) or SARS-CoV-2, diagnosis, therapeutic, and referral system of TB-COVID-19. TB status needs to be evaluated carefully, starting from patient admission, application of good management, and therapeutic strategies to prevent the rapid development and the complication of severe COVID-19 [13,51,52,53].

The following recommendations are essential for managing and treating patients with a history of MTB infection, such as LTBI or TB disease and possible SARS-CoV-2 co-infection. Firstly, community and medical teams must be aware that latent and active TB is a risk factor for SARS-CoV-2 infection, and people at high risk must be given effective preventive measures [28,43]. This vulnerable group must be monitored regularly in medical, public health, or community-based settings when resources are available. Secondly, co-infection cases must be confirmed at the admission point, and the patient needs to be placed in an isolation ward with a standardized facility. Thirdly, medical resources must be prepared for co-infected patients to anticipate the possibility of severe symptom development. Fourthly, specific therapy must be designed for cases co-infected with TB—for example, treatment with immunosuppressive agents due to the potential for LTBI activation [54].

The integrated TB control program can be carried out by preparing exceptional staff for control and treatment management. This includes aspects of diagnosis, treatment, contact tracing, outbreak investigation, latent disease, dialogue with the community and establishment of collaboration with relevant organizations. Although there is a difference between the dynamics of TB infection and COVID-19, mitigation strategies can still be carried out to aid in COVID-19 community control. Effective scientific community interactions are highly beneficial for dealing with the COVID-19 pandemic and demonstrate actions when an emergency occurs. Scientists can investigate in collaboration, with profound research funding in artificial intelligence modeling linked to clinical algorithms to predict disease severity, forming an international clinical trial platform [5].

The right timing of diagnosis is an essential factor for aligning the integration of the management of both diseases. Due to the early symptoms and acute onset of SARS-CoV-2 infection, quick detection will help early diagnosis and follow with a rapid examination of *Mycobacterium tuberculosis* infection, discovery, and radiological examinations [55]. Previous reports stated that TB has a natural effect or “weight” in increasing the probability of death among COVID-19 patients. Therefore, patient management in health services and the pathogenesis of TB and COVID-19 can be analyzed based on references to case reports to determine the basic management strategies. This also involves respiratory infections, starting from the primary or priority prevention of disease progression and the prevention of illness or spread of disease to further treat TB-COVID-19 cases in the form of a standard algorithm [28,56].

Health services and all the elemental support assisted with integrated policy need to be developed and engaged to limit the contagious spreading of COVID-19 infection and maintain the TB service program. All elements and critical support of rapid TB diagnosis must be held simultaneously, even during pandemic emergencies [17]. In a health facility, triage is vital to implement simultaneous-integrated diagnostic testing for TB and COVID-19 detection. Diagnostic testing is crucial to detect and control pathogens in public health settings, including TB and COVID-19, as the guidance for appropriate treatment informs contact tracing and decides disease control strategy. Therefore, simultaneous-integrated testing of TB and COVID-19 is urgently implemented in the TB high-burden countries. An algorithm must consider that the triage unit of a health facility needs to include parallel testing simultaneously as specimen collection and integrated testing with a multiplex diagnostic platform [45,57]. 

**Figure 1:**
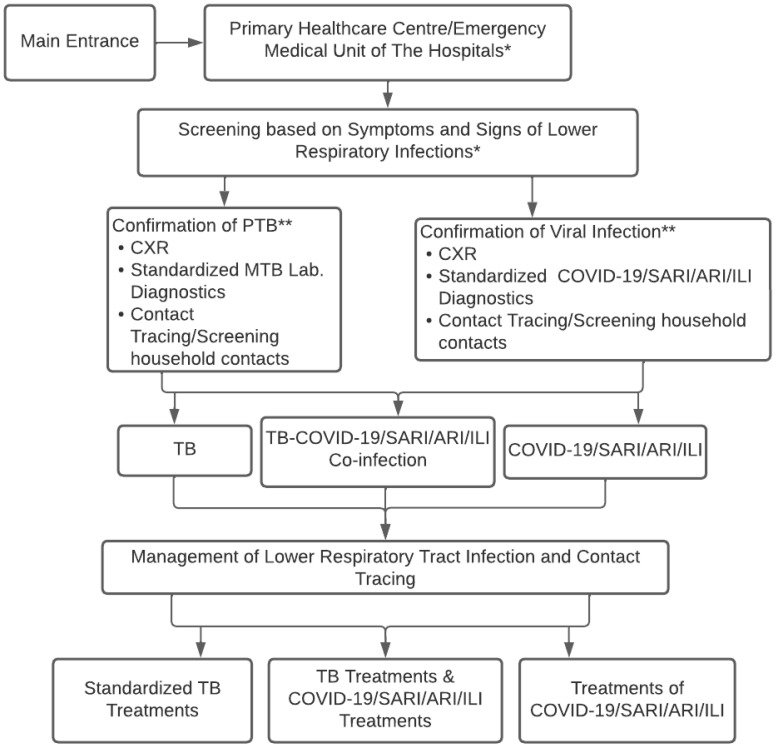
Conceptual Algorithm of TB-COVID-19 Integrated Management in Healthcare Facilities. * Main entrance admission of the patient in the primary healthcare center or emergency medical unit/clinical unit of the hospitals to implement the screening tests; ** TB and COVID-19 contacts consider universal testing for TB and COVID-19 if resources permit.

Figure 1 shows the conceptual strategies for controlling respiratory infections of TB-COVID-19/SARI/ARI/ILI to observe and design concepts appropriate to regional conditions. The concept starts from the beginning of the disease to the screening diagnosis of a “suspected” case of TB-COVID-19/SARI/ARI/ILI, as well as laboratory testing, CXR, determining diagnosis, treatment decisions, contact tracing, and infection control in the community.

**Figure 2:**
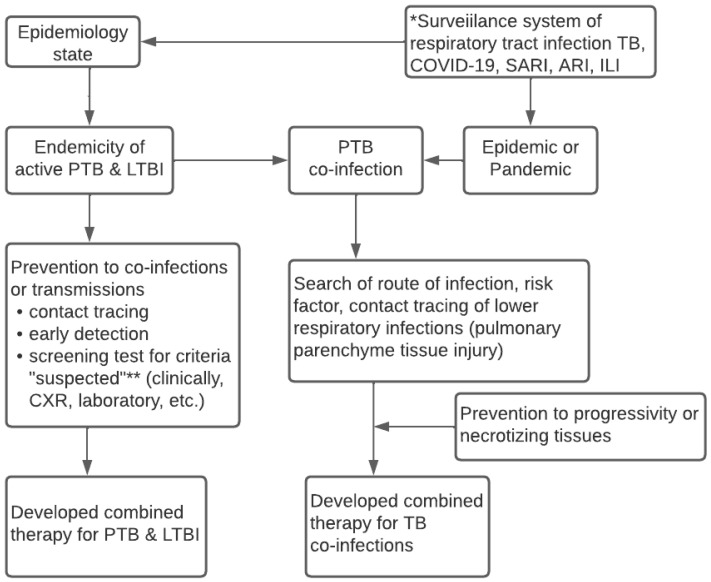
Conceptual Algorithm of TB—COVID-19 Integrated Management based on The Surveillance System. * Surveillance epidemiology system for respiratory infection related to WHO guideline 2020; ** Suspected criteria of COVID-19 based on WHO case definition 2020; ** Case definition of suspected TB based on WHO guideline 2010.

Figure 2 reveals the conceptual algorithm of the surveillance system for lower respiratory infections. It was discovered that the algorithm needs to consider the local state of epidemiologic data and carry out the integrated surveillance system of lower respiratory infections, namely TB, COVID-19, SARI, and ARI. Meanwhile, the system needs updated data on the endemicity of active TB, LTBI, and the epidemic and pandemic statuses in an area. Information on the health conditions of the local community is necessary to predict the occurrence of co-infections of pulmonary TB. The epidemiologic data or t-evidence serves as the basis to determine the design of the diagnosis, screening, and early detection, which is parallel to contact tracing. Furthermore, it is essential to prevent progressivity, transmissions, and decisions from appropriately combined therapeutics.

Based on these results, future implementation needs to involve all health services to integrate the governance of TB-COVID-19 and comorbid. Implementing the TB-COVID-19 integration algorithm does not rule out ILI, ARI, and SARI or ignore HIV, comorbid diabetes mellitus, and other immunocompromised patients, as shown in Figure 2. Every time a patient with symptoms of respiratory tract infection visits a health care unit, a standardized management algorithm for patients with suspected PTB, PTB co-infection of COVID-19/SARI/ARI/ILI is carried out to implement the standardized prevention, treatment, patient care, and control of infectious diseases in the community. Similarly, it involves the standard flow of patient management at every regular visit of PTB patients in the Health care unit. The integrated treatment of TB-COVID-19 must also consider information from WHO 2020, TB, and COVID-19 treatment guidelines.

Implementing the algorithm of TB-COVID-19 integrated management could achieve good patient care with outcomes decreasing the morbidity and mortality of respiratory tract infections and contribute to TB control, especially in endemic countries.

## 5. Conclusions

TB is more complicated and crucial to managing when co-infection with COVID-19/SARI/ARI/ILI or other respiratory viral infections. Therefore, based on implementation research and multicentre design, there is a need to create an updated guideline and algorithm for the integrated management of TB-COVID-19/SARI/ARI/ILI.

## Data Availability

Not applicable.
